# The relationship between university student help-seeking intentions and well-being outcomes

**DOI:** 10.3389/fpsyt.2024.1407689

**Published:** 2024-07-05

**Authors:** Melinda McCabe, Michelle Byrne, Judith Gullifer, Kim Cornish

**Affiliations:** ^1^ Turner Institute for Brain and Mental Health, Faculty of Medicine Nursing and Health Sciences, Monash University, Clayton, VIC, Australia; ^2^ School of Psychological Sciences, Faculty of Medicine Nursing and Health Sciences, Monash University, Clayton, VIC, Australia

**Keywords:** mental health, help seeking, wellbeing, university student, higher education

## Abstract

University represents a time of both great uncertainty and change as well as a time of opportunity and learning. University students represent a population both at a higher risk of experiencing poor mental health and diagnosis and a population with relatively greater access and communication of both mental health literacy resources and mental health support services. Despite this, we consistently see low intention of help-seeking for mental health services or health services, with a clear preference shown for personal contacts such as friends or parents. To understand help-seeking intentions and their relationship with well-being, the current study explored two core hypotheses, when assessing a broad range of help-seeking options, the likelihood of seeking support from a range of sources will cluster together to create help-seeking groupings that can be further explored (1) and that across these help-seeking factors, there would be difference in intention score across students who fall within “at-risk,” “low,” or “normal to high” well-being groupings (2). Through a series of exploratory factor analysis (EFA) on a subsample (*N* = 178) and final confirmatory factor analysis (*N* = 1597) identified five help-seeking factors: Intimate Partner (single item), Personal Relations (friends, parents, and other relatives), External Health Service (external mental health provider and health provider), University Health Service (university mental health and health provider), and Digital and Distal Professional (digital apps, websites or forums, telehealth, religious leaders, and phone or online emergency services). To address hypothesis 2, a multivariate analysis of covariance was run to assess help-seeking intentions across factors between students with “at-risk” (*N* = 453), “low” (*N* = 484), or “normal to high” (*N =* 563) well-being scores. Although significant differences were found between groups on almost all help-seeking factors (except External Health Service), the differences between groups were small. However, consistently those with “normal to high” well-being demonstrated higher intention to seek help compared to “low” and “at-risk” groups. Across all groups, Personal Relations demonstrated the highest average help-seeking intention score and, in addition to supporting findings in previous literature, represents a potential “lowest hanging fruit” of help-seeking source for university students.

## 1 Introduction

After 12 years of structured and consistent classroom learning, the transition from secondary education into a university environment can be challenging. Often accompanied by newly found independence and responsibility, it is no surprise that such a large proportion of university students reported high levels of stress, anxiety, and feelings of loneliness and depression (1) when transitioning to tertiary education. To combat this, universities fund, provide and promote discounted or free counseling and psychological support to their students. Despite the high prevalence of universities now offering this support, it remains unclear if the uptake and utilization are meeting the needs of their student communities nor is it understood how and from whom university students seek help when they are struggling with their mental health and well-being.

Throughout this paper, we will refer to both undergraduate (known in Australia as a bachelor’s degree) and postgraduate students as university students. Most university students begin their journey of higher education in the early adult years between 17 and 25 years old. This particular age group has already been highlighted as an at-risk age for the development of mental health diagnosis (2). Such diagnosis, particularly at a very young age, can have extensive and pervasive outcomes on an individual’s prognosis, quality of life, and their overall well-being. Well-being is defined as the quality of life and the ability for an individual to contribute and be part of wider society with a sense of meaning and purpose (3). Although onset rates and measures of disease prognosis are critical elements of understanding, it only represents individuals who have reached the point of diagnosis. Diagnosis inherently requiring ongoing or long-term symptoms, significant changes to their ability to function and complete necessary daily tasks, and consultation and ongoing engagement with a health or mental health professional. Not all individuals suffering from poor well-being or mental health meet these criteria for diagnosis. To account for these individuals and better understand the broader impact of low well-being and poor mental health, it is important to understand the pre-clinical impact through non-clinical concepts such as well-being or stress.

The fields of psychology and social sciences often understand mental illness prevention as an educational task, working with communities who may be at high risk of developing mental illness to promote awareness and literacy programs, with the goal of providing the community with the tools they need to identify and change situations and behavior early, before it impacts health. Unfortunately, education today is not the same as this important mental health education. Even highly educated populations in some of the wealthiest countries in the world see a lack of health literacy amongst their adult population (4), often disproportionately prevalent amongst those that have a lower income, are already unwell, and are already demonstrating poor health behaviors that are likely to worsen quality of life and physical health over time. Although a seemingly simple solution, broadly implemented mental health education faces significant challenges in the development and delivery of effective and timely mental health tools that will prevent illness onset and improve, measurably, the well-being of a community. The university student population is unique, representing an at-risk group who is also relatively easy to access and communicate with, all culminating through a single (or cluster) of physical and online locations. Because of this, they are an ideal population to begin to build working frameworks and guidelines that can then be expanded into new communities.

Within the population of university students, many higher education institutions have taken up the responsibility of developing and delivering preventative interventions for their students at either no, or low cost, often taking the form of free counseling services or information sessions. Across many countries and universities, it seems that these services are proving useful and beneficial to the students who choose to engage, whether for mental health concerns or academic concerns (5–7), even during the COVID-19 pandemic (8, 9). For many, if not all of the students using these services, they are not currently diagnosed with mental illness and are seeking preventative support for academic, stress/anxiety, or relationship concerns (10) where some students may also be seeking support due to self-diagnosis or suspicion of meeting the criteria for diagnosis (11). The COVID-19 pandemic has also seen an increase in the use of online or remote tools for mental health support, demonstrating an escalation in the development and delivery of preventative tools (12) including smartphone apps or tools and information easily accessible through app-based or web-based platforms. Despite this focused provision of resources, it remains unclear how effective these new digital tools and apps are compared to traditional counseling and face-to-face methods (13), and how likely these populations are to utilize and use these services when they are needed.

Intentions, the goal or purpose of someone’s thoughts and behaviors, are outlined by the Theory of Planned Behavior (TPB, 14) as the central factor of predicting and influencing behavior. Encapsulating the motivational factors further outlined by the TPB including attitudes, subjective norms, and perceived behavioral control, intention describes how much time, effort, and energy a person is willing to exert to engage in any specific behavior. The General Help-Seeking Questionnaire (GHSQ; 15) is a two-item scale focusing on the intention of a participant to seek help from a range of sources in different circumstances. This scale uniquely provides a range of potential help-seeking sources to the participant to better understand help-seeking intentions across friend or parent, to health professional or helpline. Although developed almost two decades ago, Wilson (2005) and colleagues identified consistent trends in different likelihood of help seeking across these options, with friends and personal contacts showing higher likelihood of help seeking compared to health professionals. This preference remains when exploring reported barriers of professional help seeking amongst college students (16) who commonly report a preference for personal relationships for support, or a preference for self-support and unwillingness to seek help from any source (17). The TPB in its ability to predict future behaviors does not explicitly name current well-being or emotional state as a predictor of intention and later behavior; however, in practice, research literature has begun to highlight a potential impact. In 2016, Goodwin and colleagues explored the relationship between well-being and help seeking amongst first year university students in Ireland for whom university provided support was available. Those with higher levels of mental health and well-being were more likely to seek support from informal sources such as friends or family, and those with lower levels of well-being were not likely to seek help from any source whether formal (medical professional) or informal. Observations of this concerning trend on help seeking and well-being remain consistent over both older and newer research (17–20). Research highlighting these findings all note the same concern and worry about these observations but fluctuate in terms of understanding the help seeking source this worry relates to. There could remain some options for help seeking that could act as a “lowest hanging fruit” of support, a source or group of sources that could be promoted as a gateway method of help seeking for those who are struggling the most and least likely to reach out to more traditional mental health support.

Understanding how, when, and if a university student seeks help from any source, in relation to their well-being at the time, is a critical first step to building and deploying preventative mental health solutions that could increase rates of future help seeking from any source in the future. As such the current study has two aims: to explore help-seeking intentions amongst university students using a range of help-seeking options relevant to what is currently offered in a university setting and within the age group of university students (1) and to explore how well-being impacts the intention to seek help from a range of providers whilst controlling for perceived stress that may fluctuate over time (2). Subsequently, it is hypothesized that the likelihood of seeking support from a range of sources will cluster together to create help-seeking groupings that can be further explored (1). It is also hypothesized that there will be a difference in help-seeking intentions across these grouped sources for students who fall within the “at-risk,” “low,” or “normal to high” well-being groupings (2).

## 2 Materials and methods

### 2.1 Participants

The participants for the current study include independent responses from 1,776 university students enrolled at a Monash Campus. This dataset is part of a larger dataset of 2,151 responses over the course of four 2022 Monash Thrive (THRIVE@Monash) surveys throughout May (*N* = 861), July (*N* = 594), October (*N* = 606), and December (*N* = 498) (21–24). In 2022, the total Monash student enrollments across Australian campuses were 72,154, including full- and part-time students (25), meaning this sample represents only 2.4% of the total student enrollments at the time. Only the first response was retained after assessing the reliability of all variables (described in data analysis). Therefore, the final sample consisted of 1,776 independent responses in 2022. Of the final sample, 63.8% identified as female, the average age of this sample was 25.02 years (*SD* = 8.59), and this was consistent across all time points. This study was approved by the Monash University Human Research Ethics Committee (ID: 32546).

### 2.2 Materials

#### 2.2.1 Demographics

All demographics were collected at the beginning of the survey and included variables such as age (in years), gender identity (female, male, non-binary/diverse, prefer not to say, and open text response alternative), year level (first, second, third, or fourth and higher), current enrolled location (country and campus), and if they are currently studying away from campus location (binary yes/no).

#### 2.2.2 Help-seeking intentions

Due to the preventative mental health nature of the study and research questions, we only used item 1 from the GHSQ (15) to measure help-seeking preferences where item 2 refers to help-seeking when experiencing suicidal ideation. Item 1 asks participants to rate how likely they are to seek help from a series of individuals and health providers if they were experiencing an emotional or personal problem. Participants respond to each prompt on a 7-point Likert scale of “extremely unlikely” (1) to “extremely likely” (7) with a middle option of “neither likely nor unlikely” (4). The standardized list of prompts includes Intimate Partner, Friend, Parent, Other Relative, Mental Health Professional, Phone Helpline, Doctor/GP, Minister, or Religious Leader, I would not seek help, I would seek help from others (open text response addition). The current study adapted the original list to include additional sources of mental health support provided by, and promoted by, the university including [university name] mental health provider, and [university name] GP/Doctor, Website or Forum, and Digital Health App. This study removed “would not seek help” and “would seek help from other source” options where “would not seek help” caused confusion for participants during design phase, and “other source” required an open text response that was out of the scope of the original research plan. These changes resulted in a total of 13 help-seeking items. The alpha for the original GHSQ is α = .70. The alpha for this study and the adjusted GHSQ is α =.84.

#### 2.2.3 Well-being

The current study utilized the World Health Organization 5-item well-being scale (WHO-5; 26). Using a 6-point Likert scale from “at no time” to “all the time,” the WHO-5 measures the frequency of experiencing markers of holistic well-being in the 2 weeks prior. These markers of well-being include “I have felt cheerful in good spirits” and “My daily life has been filled with things that interest me.” Once rated on a scale of 0 (at no time) to 5 (all the time) scores are summed and multiplied by 4 to provide a final score between 0 (absence of well-being) and 100 (maximum well-being). The reported mean of the WHO-5 is 50 based on development and validation findings, where scores less than 28 are reported in literature as being particularly at risk of major depression (26). The alpha measured for the WHO-5 in the current study is α =.90. The current study uses the WHO-5 scores to create and compare three well-being groups: “at risk” (scores less than 28), “low” (scores between 29 and 49), and “normal to high” (scores of 50 or higher).

#### 2.2.4 Perceived stress

The current study uses the 10-item Perceived Stress Scale (PSS-10; 27) to measure stress at all time points of data collection to control for fluctuations in stress across time points during and post-teaching semester. The PSS-10 uses a 5-point Likert scale (Never = 0 to Very Often = 4) to assess participant responses to prompts such as “how often have you been upset because of something that happened unexpectedly” and “how often have you felt nervous or stressed” over the course of the month prior to responding. Of the 10 items four are positively phrased and reverse scored (items 4, 5, 7, and 8), all items are then summated to provide a total score. PSS-10 scores range from 0 to 40 and are often clustered in groups of low, moderate, or high stress. For the purpose of this study, the PSS-10 score is used as a continuous control measure and was not grouped. The alpha measured for the PSS-10 in this study was α =.87.

### 2.3 Procedure

All measures are anonymously self-reported online through Qualtrics survey. Participants were invited to participate through central communications (direct email) from university leaders and social media advertisements. They completed all surveys within a 2-week period after indicating that they were over 18 years of age, provided informed consent, and indicated that they were enrolled at the university. Each survey required approximately 20 min to complete online; no compensation or prizes were offered for completion of the survey. Two surveys (May and October) took place during the final two teaching weeks of semesters 1 and 2, whilst the other two surveys (July and December) took place outside of teaching semesters post-examinations. Stress was included as a controlling factor in analysis to assist in controlling for how academic and related stress may have fluctuated across time points.

### 2.4 Data cleaning and analysis

All data analysis was undertaken using R (28) and RStudio (29) using packages “psych” (30) and “lavaan” (31). The sample used for this study was sourced from a larger dataset of 2,151 responses across May *N* = 861, July *N* = 594, October *N* = 606, and December *N* = 498. The current sample is the first response only from each individual participant, identified by a pseudo-identifier using participants initials and the last four numbers of their phone number at the start of each survey. This identifier was used to track responses over time and to ensure only the first response from each participant was included in this study. To ensure first responses from participants were representative, for each variable of interest, we calculated the intraclass correlation coefficient to assess how much variance was attributable to within-person variance, as well as Cronbach’s alpha to assess reliability. All variables had a moderate or high cronbach’s alpha ensuring that responses over time were consistent where multiple responses could be therefore removed to retain the integrity of the data as cross-sectional. Data were included if participants completed all items of interest and, due to the large sample size, no missing data were imputed.

To test hypothesis 1, a series of data-driven EFA analysis was run to cluster help-seeking sources into distinct groups based on current data and the current sample. Due to the large sample size smaller subsample was created for EFA analysis, this test sample consisted of a randomly selected 10% of the total sample (*N* = 178). The remaining 90% of the sample was retained as a final sample (*N* = 1598) used in the confirmatory factor analysis (CFA). To address hypothesis 2 a multivariate analysis of covariance (MANCOVA) was conducted comparing the help-seeking intention scores across the groups of help-seeking sources finalized through CFA analysis across different levels of well-being. The levels of well-being acted as the grouping variables, split into “at-risk” well-being, “low” well-being, and “normal to high well-being” based on the scale manual and previous literature (32). As the sample is collected across four time points throughout 2022 the MANCOVA analysis will control for perceived stress as a measure that may have fluctuated over time based on proximity to high-stress points such as exams and assessments.

See **Table 1** for the demographic data representing both the test model sample (EFA) and the final model sample (CFA and MANCOVA).

**Table 1:** Available demographic statistics for test model sample and final model sample.

	Test Model (10% subsample)			Final Model (90% subsample)		
	Frequency	Percentage (%)	N^a^	Frequency	Percentage (%)	N^a^
Gender Identity						
Female	114	64.0	178	1019	63.8	1597
Male	56	31.5		506	31.7	
Non-binary or gender diverse	5	2.8		49	3.1	
Other or prefer not to say	3	1.7		23	1.4	
Demographics						
Timepoint 1	80	44.9	178	602	37.7	1597
Timepoint 2	37	20.8		352	22.0	
Timepoint 3	29	16.3		380	23.8	
Timepoint 4	32	18.0		263	16.5	
International student	71	39.9	178	669	42.0	1593
Residential student	26	14.6	178	241	15.2	1591
Undergraduate enrolled	117	65.7	178	1024	64.2	1594
First year of degree	65	36.5	178	550	34.4	1597
Second year of degree	52	29.2		451	28.2	
Third year of degree	26	14.6		302	18.9	
Fourth year or higher of degree	35	19.7		294	18.4	
Higher degree by research	30	49.2	61	293	51.4	570
Currently working	107	60.11	178	856	53.6	1597
Enrolled in Australia	176	98.9	178	1563	98.0	1595

a Total sample size for EFA analysis using Test Model was *N=178*, Total sample size for Final Model CFA and MANCOVA was *N=1598*.

A demographic breakdown of the help-seeking intention findings between the test model and final model can be found in **Table 2**.

**Table 2:** Help seeking intention descriptives across test and final model samples.

	Test Model (10% subsample)			Final Model (90% subsample)		
	Frequency likely to seek help	Percentage (%) likely to seek help	Total	Frequency likely to seek help	Percentage (%) likely to seek help	Total
Adjusted General Help Seeking Questionnaire						
Intimate Partner	113	63.5	178	932	58.3	1598
Friend	137	77.0		1185	74.2	
Parent	94	52.8		919	57.5	
Other Relative	64	36.0		566	35.4	
University Mental Health Provider	60	33.7		455	28.5	
External Mental Health Provider	100	56.2		677	42.4	
Phone or Online Emergency service	35	19.7		289	18.1	
University General Practitioner	52	29.2		364	22.8	
External General Practitioner	89	50.0		641	40.1	
Minister or Religious Leader	12	6.7		133	8.3	
Medical Professional through Telehealth	54	30.3		425	26.6	
Mental Health Smartphone Apps	31	17.4		327	20.5	
Websites or online forums	50	28.1		376	23.5	

## 3 Results

### 3.1 Exploratory factor analyses

To condense the responses where possible for further analyses, we first fit a total of three EFA models to explore latent factors of help-seeking intention variables using the 10% test model sample. For all EFA analysis, Maximum Likelihood Factor Extraction Method was used and a minimum factor loading is considered .32 (as per 33).

EFA 1: The initial EFA included all 13 prompts, five factors were set based on previous literature (15). Findings from EFA 1 found one prompt, “Intimate Partner,” that did not load onto any factors. Due to these findings, “Intimate Partner” was removed from further EFA analysis and was analyzed in final analysis as a separate variable.

EFA 2: The second EFA was run without “Intimate Partner” with five factors set. These findings showed all prompts loading onto factors; however, one prompt, “Phone or Online Emergency service” loaded onto a factor alone. As there should ideally be a minimum of two variables to create a factor (34), a third EFA was run, removing a factor.

EFA 3: The third EFA retained all prompts except “Intimate Partner” and set four factors. The third EFA findings showed all prompts loading onto the four factors, the factors and their loadings can be found in **Table 3**. All details of the three EFA can be found in **Appendix A**.

**Table 3:** Final EFA factors and their loading.

Factor	Prompt name	Loading †
Factor 1 (Personal Relations)	Friend	0.409
	Parent	0.820
	Other Relative	0.724
Factor 2 (External Health Professional)	External Mental Health Professional	0.891
	External General Practitioner	0.563
Factor 3 (University Health Professional)	University Mental Health Professional	0.573
	University General Practitioner	1.081
Factor 4 (Digital and Distal Professional)	Phone or Online Emergency Service	0.368
	Medical Professional through Telehealth*	0.440
	Mental Health Smartphone Apps	0.816
	Websites or online forums	0.843
	Minister or Religious Leader	0.394

†A minimum loading score of.32 was used for reported loading.

*Also loaded onto factor 2 at 0.337, highest loading was retained.

The final model also showed the best fit and was retained for further CFA analysis. Fit indices from the final EFA 3 can be found in **Table 4**. Continued analysis will name Factor 1 “Personal Relations,” Factor 2 “External Health Professional,” Factor 3 “University Health Professional,” and Factor 4 “Digital and Distal Professional.”

**Table 4:** Final model EFA goodness-of-fit indicators of help seeking intention factors, N=178.

Model	X^2^	p-value	df	ECVI	SRMR	MFI	CFI	RMSEA
4-factor	53.45	.001	24	0.907	0.031	0.921	0.966	0.083

### 3.2 Confirmatory factor analysis

**Figure 1:**
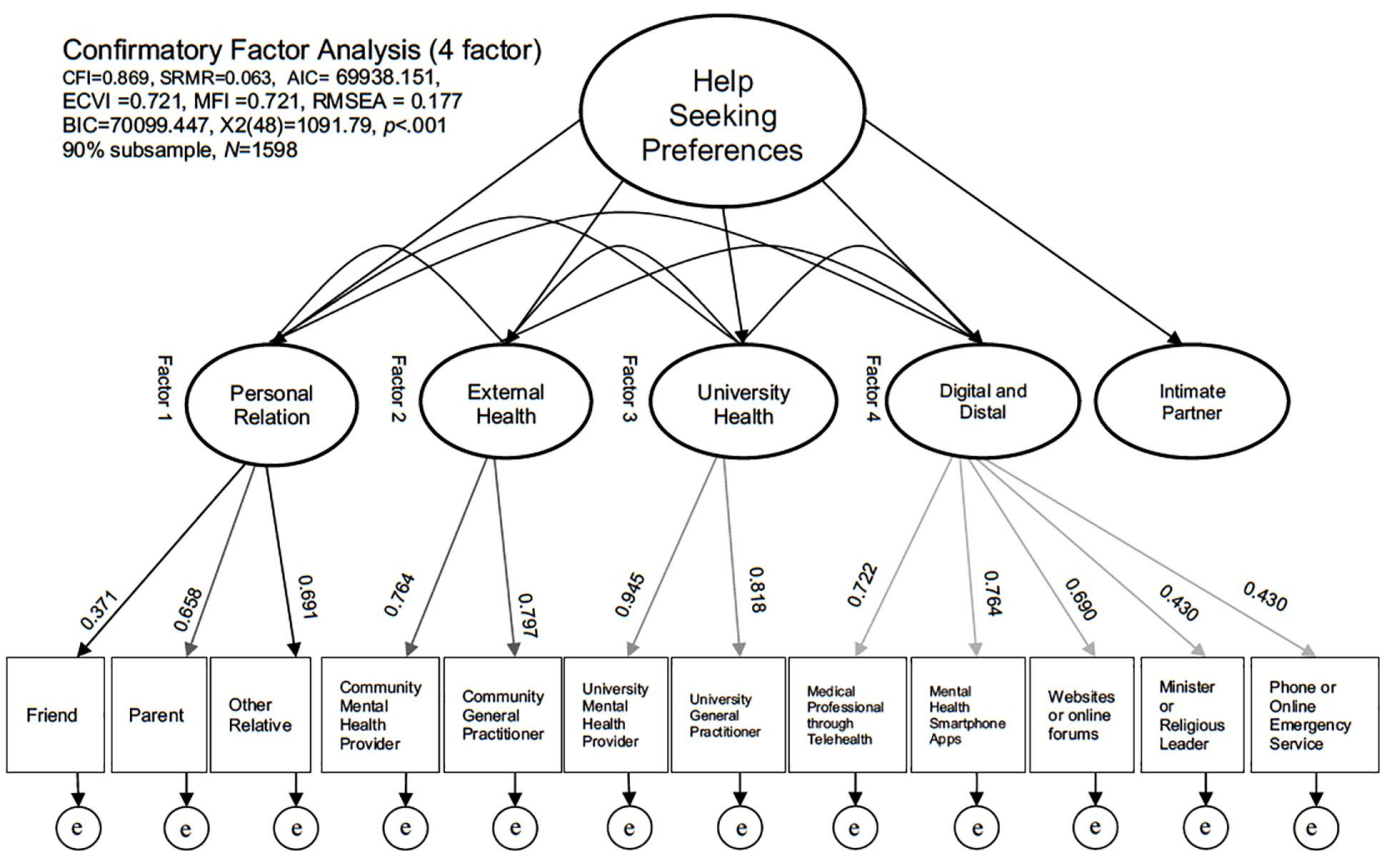
CFA finding for help seeking intention factors using a 4-factor model, N=1598.

Using the model found in the fourth and final EFA analysis, a further CFA analysis was run using the 90% final model sample. For findings from CFA analysis, see **Figure 1**.

### 3.3 Multivariate analysis of covariance

**Figure 2:**
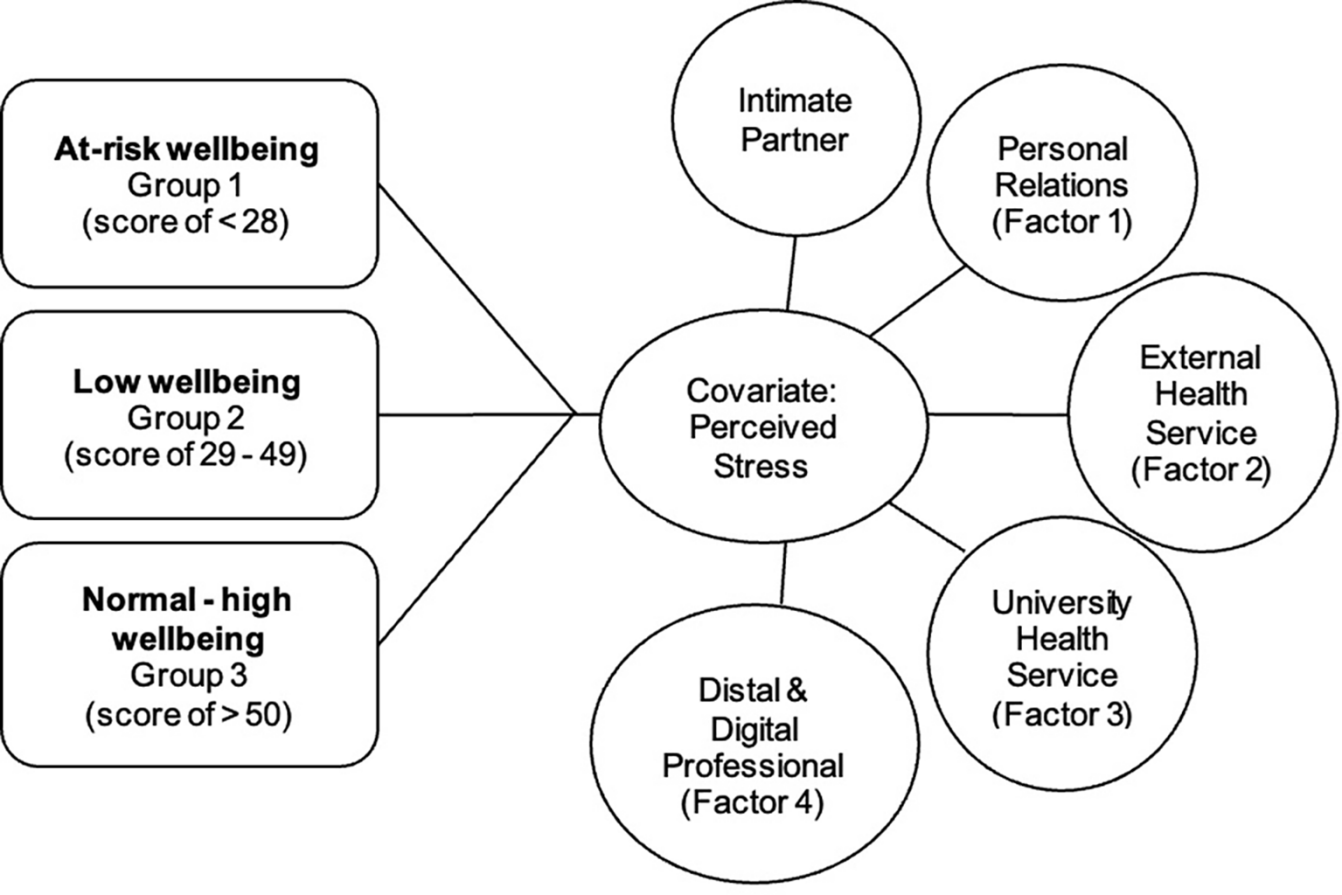
Visual Representation of MANCOVA analysis run comparing help seeking intentions across wellbeing groupings whilst controlling for perceived stress.

Using the model outlined in **Figure 2**, a MANCOVA analysis was run to compare the difference in help-seeking intentions across university students experiencing “at-risk” (between 0–28, *N* = 453), “low” (between 29 and 49, *N* = 484), or “normal to high” (between 50 and 100, *N =* 563) well-being whilst controlling for perceived stress. Assumption testing for MANCOVA analysis was completed as per Tabachnick and Fidell (33).

Multivariate normality for all dependent variables was assumed based on Q-Q plots, assumptions of linearity for all dependent variable pairings and covariate-dependent variable pairings were assumed based on correlational analysis, all univariate and multivariate outliers were identified using Mahalanobis Distance (threshold of 20.5), only five outliers were identified; however, as the relevant Cooks Distance was less than .01, all outliers were retained. Box’s M was used to measure homogeneity of variance–covariance; this assumption was violated according to Box’s M significance (<.001); however, due to the sensitivity of Box’s M in large samples, the analysis was continued using Pillai’s trace to account for this assumption violation (35).

Descriptive statistics for help-seeking intentions across the three well-being groups can be found in **Table 5**.

**Table 5:** Help seeking intention descriptive statistics across each wellbeing group (mean, standard deviation) for both raw average scores and factor generated score.

	At risk		Low		Normal to high	
	Raw average	Factor score	Raw average	Factor score	Raw average	Factor score
Personal Relations (factor 1)	3.79 (1.46)	-0.18 (0.51)	4.20 (1.39)	-0.03 (0.50)	4.82 (1.27)	-0.07 (0.47)
External Health Service (factor 2)	3.78 (1.78)	-0.07 (1.34)	3.91 (1.68)	0.04 (1.26)	3.73 (1.68)	0.02 (1.33)
University Health Service (factor 3)	3.01 (1.66)	-0.11 (1.59)	3.05 (1.58)	-0.10 (1.50)	3.30 (1.68)	0.17 (1.63)
Distal and Digital Professional (factor 4)	2.49 (1.19)	-0.15 (1.14)	2.65 (1.25)	-0.02 (1.16)	2.83 (1.37)	0.14 (1.28)
Intimate Partner	4.25 (2.31)	–	4.77 (2.13)	–	5.21 (1.98)	–
Stress (covariate)	26.62 (5.75)	–	22.02 (5.25)	–	16.77 (5.30)	–

Findings of the MANCOVA analysis showed there was a significant effect of well-being grouping on the combined outcome variables of help-seeking intentions, using Pillai’s trace *F*(10, 2928) = 9.60, *p* <.001, partial *η* ^2^=.032.

Analysis of the help-seeking intention groups individually demonstrated a difference between well-being groups for all help-seeking intention outcomes aside from External Health Service (factor 2); for all results for each individual help-seeking intention outcome, see **Table 6**.

**Table 6:** Individual outcome statistics for help seeking intention outcomes.

	Degrees of freedom	F statistic	Significance (p-value)	Effect size (partial *η* ^2^)
Intimate Partner	2, 1467	10.53	<.001	.014
Personal Relations (factor 1)	2, 1467	27.49	<.001	.036
External Health Service (factor 2)	2, 1467	1.92	.146	.003
University Health Service (factor 3)	2, 1467	14.83	.002	.008
Distal and Digital Professional (factor 4)	2, 1467	11.01	<.001	.010

Further *post-hoc* analysis for all significant outcome variables using Games–Howell to account for unequal homogeneity of variances can be found in **Table 7**.

**Table 7:** Games-Howell post-hoc analysis of outcome variables across wellbeing grouping.

	Group Level 1	Group Level 2	Mean difference	Significance (p-value)
Intimate Partner	At risk	Low	-.53	<.001
	At risk	Normal	-.95	<.001
	Low	Normal	-.42	.004
Personal Relations (factor 1)	At risk	Low	-.15	<.001
	At risk	Normal	-.36	<.001
	Low	Normal	-.20	<.001
University Health Service (factor 3)	At risk	Low	<-.001	.999
	At risk	Normal	-.30	.009
	Low	Normal	-30	.006
Distal and Digital Professional (factor 4)	At risk	Low	-.13	.199
	At risk	Normal	-.31	<.001
	Low	Normal	-.18	.042

## 4 Discussion

The current study explored two hypotheses. The initial hypothesis explored how students’ intentions to seek help from different sources may cluster together into help-seeking factors (1) for further exploration. The second hypothesis explored the difference in help-seeking intentions across these help-seeking factors based on well-being grouping whilst controlling for stress. The first hypothesis was explored through a series of EFA to understand how differing help-seeking sources may cluster together based on university students’ intention to use these services if they were struggling personally or emotionally. Three EFA were completed with four distinct factors identified and confirmed through confirmatory factor analysis after the removal of “intimate partner” from the factor model. The four distinct factors identified included “Personal Relations” (including Friends, Parents, Other relatives), “External Health Service” (including External Mental Health Provider and External Health Provider), “University Health Service” (including University Mental Health Provider and University Health Provider), and “Digital and Distal Professional” (including Digital app, Website or Forum, Medical Professional through Telehealth, Minister or Religious Leader, and Phone or Online Emergency Service). The second hypothesis was explored using a multivariate analysis of variance and covariance (MANCOVA) where well-being scores were used to group participants into either ‘at-risk’, ‘low’, or ‘normal to high’ well-being, perceived stress was used as a covariate to control for changing stress due to the multiple time points of data collection and varying academic conditions over the year, and the outcome variables of interest were the help-seeking intention factor scores identified and created in hypothesis 1. Overall MANCOVA findings highlighted a difference in help-seeking intentions across all well-being groups for Intimate Partner, Personal Relations, University Health Service, and Digital and Distal Professional however it was non-significant for Community Health Service. Further *post-hoc* analysis demonstrated consistent significant differences in help-seeking intentions across factors for all well-being groups aside from the ‘low’ and ‘normal’ well-being group on Intimate Partner, University Health Service and Digital and Distal Professional, no difference between groups (with alpha set and .001) for University Health Services, and only a difference between ‘at-risk’ and ‘normal’ well-being groups for Digital and Distal Professional. Throughout all help-seeking source factors it was shown that those in the lower well-being groups were consistently less likely to ask for help compared to those in the ‘normal to high’ well-being group.

### 4.1 Help-seeking intention factors

Initial EFA highlighted ‘Intimate Partner’ as a help-seeking option that did not load onto any other factor despite a potential theoretical connection to the ‘Personal Relations’ factor which included friends, parents, and relatives. A preference for help-seeking from personal sources, particularly peers and friends, has been consistently highlighted in previous literature (15, 16, 36) and it is an interesting observation that intimate partners may not be considered part of this ‘personal relations’ group when it comes to help-seeking intentions. This observation may reflect a more complex relationship with intimate partners compared to friends or relatives, particularly when seeking support for personal or emotional difficulties. Previous studies highlight that in younger age groups intimate partner relationships can often be the cause of mental health or emotional concerns (37) and was highlighted as the fifth most common concern for adolescents in counseling services. The age of the sample also highlights a likely ‘new’ or inexperienced and less serious nature to relationships which may reduce likelihood of seeking help from their intimate partner. A supplement paper published in 2007 (38) suggests family members may be a more important source of help in younger years, where as young people age seeking help from friends or intimate partners becomes more important later in life. It should be noted that this study did not verify if a participant was in, or had been in, an intimate relationship, therefore these findings may also be skewed by a difference in preferences for those in a relationship as compared to those who were imagining themselves in a relationship when responding to the item. The important distinction of help seeking from partners does highlight a need for further focus in this area particularly for young people who may be in their first relationship or are going through significant relationship milestones for the first time, and how this relates to their willingness to seek help from their partner when in need.

Another key observation of the multiple factor analyses was the clear distinction between external and university-provided health services throughout every iteration of EFA and confirmed in the CFA analysis. This finding is consistent with previous literature, particularly mixed method and qualitative literature, that highlights barriers and concerns for seeking help from their university counseling or mental health service (10) including but not limited to unhelpful beliefs that a service overwhelmed or would require extensive waiting periods, being unaware of the support and elements like privacy and confidentiality surrounding the support provided, or concerns that these support services won’t help them either based on previous experiences, unhelpful beliefs or testimony from their peers.

Finally, after removing personal relations, and separating external and university support networks this model clustered all ‘other’ help-seeking options into the Digital and Distal Professional factor. This factor seems to represent the relatively novel and emerging help-seeking options including all of the non-face-to-face help-seeking options such as telehealth. Telehealth options are increasingly popular particularly during and post- the COVID-19 pandemic and remain practical and necessary for those unable to travel to inner city locations to seek help. However, accessibility in terms of the number of providers trained and available to provide the service, potential cost if not covered by insurance or healthcare services, and a consistent preference for face-to-face appointments, particularly for mental health appointments, have all been highlighted as barriers to using telehealth services (39). Of key interest to this study is the relatively low interest in help-seeking from digital sources. Often cited in mission statements from the developers of mental health apps and websites, the provision of affordable, accessible, and secure mental health support is the goal of these digital options. Despite this, these findings still show that only approximately 20% of students would seek help from either a mental health app or mental health website or forum. The question remains if and how digital solutions may have a more permanent presence in the mental health space, with many also unsure if they should be in the mental health space at all. The low rates of intention to use in the current study suggest more work needs to be done in understanding if and why university students would use and benefit from digital tools for their mental health and well-being particularly from a non-clinical and preventative perspective.

### 4.2 Help-seeking intentions across well-being groups

The second aim was explored through a MANCOVA comparing well-being groups of at risk, low, and normal to high on their help-seeking intentions across the four factors and intimate partner whilst controlling for perceived stress. It should be noted that some assumption testing was violated prior to analysis and therefore results should be interpreted accordingly. The overall MANCOVA analysis showed a significant difference between the three well-being groups when taking into account all outcome variables of help-seeking intentions. A visual observation of average help-seeking scores indicate that those in the at-risk well-being group consistently demonstrated the lowest help-seeking intentions across all help-seeking factors and intimate partner. This was followed by the low well-being group having the second lowest average help-seeking intention and the normal to high well-being group had the highest average help-seeking intention (for all factors aside from External Health Services). A similar finding was highlighted in a 2016 paper (36) amongst an Irish student population where those with low/average well-being, as measured using the Warwick-Edinburgh Mental Wellbeing scale (40), were shown to be the least likely to seek help overall compared to those with higher scores.

When comparing all help-seeking intention outcome variables separately across the three well-being groups a significant difference was found for all except External Health Services where the average raw score for all three groups fell closely to the “slightly unlikely” response for the GHSQ with a very similar average and standard deviation. It should be noted that the average raw score difference on almost all factors often indicated a change between either slightly unlikely and neither likely/unlikely, or neither likely/unlikely and slightly likely to seek help; it remains to interpretation how this could change the eventual help-seeking behavior of a university student as their well-being changes over time.

Further *post-hoc* findings highlighted a significant difference in only some of the help-seeking source factors, specifically only the “at-risk” group showed significant differences with the other well-being groups for seeking help from an intimate partner, similarly only the comparison between “at-risk” and “normal” showed significant differences for Digital and Distal Professional. This difference across the two groups, when compared to average score responses, still highlights a high reluctance to seek help from Digital and Distal Professionals, although potentially skewed by components of this factor such as “Religious Minister or Leader” that showed very low percentage likely to seek help from. Despite this, these findings demonstrate that there is much more to do in this space to improve the likelihood of University students to uptake and use digital options. University Health Service showed no significant differences between groups and in contrast, there was a significant difference across all groups for Personal Relation. Although at first glance this may indicate that seeking help from University services could be the “low-hanging fruit of help seeking,” where the comparison between “at-risk,” “low,” and “normal to high” well-being is only non-significant throughout groups for University health services; however, this will require further investigation where this could mean no difference between all groups being generally willing or generally unwilling to use these services.

### 4.3 Limitations of the current study

The current results should be interpreted with appropriate caution to reflect some of the methodological and statistical limitations of the findings. Methodological limitations include the method of data collection through large-scale anonymous surveys across four time points in 2022, alongside the restriction of quantitative data only, including the removal of the open text option of “other help-seeking option” within the GHSQ. It should also be noted that this study utilized only a short (20 min), anonymous, and non-compensated data collection model which may have impacted not only the quality of results obtained but also restricting the number of potential participants willing to take part. These data although able to assess the responses of a large sample size do not provide the qualitative meaning and context behind the help-seeking intention responses. In addition, without the clear and meaningful collection of data pertaining to relevant stigma, bias, or other physical limitations to seek help we are unable to extrapolate the causal context behind why some students were unlikely to, or likely to, seek help from any of the options provided. This is something that should be explored carefully and with diverse data collection methodology to ensure we understand the “why” behind these quantitative summaries. It should also be noted that statistically the MANCOVA analysis in hypothesis should be interpreted with caution due to some violations of the MANCOVA assumption testing; however, this has been controlled for where possible. Further interpretation of significant differences between groups should also be interpreted with caution as some significant findings only represent slight differences between help-seeking intentions across groups; this has been highlighted in all findings summaries. Future research may incorporate longitudinal measures of help-seeking intention or the inclusion of longitudinal help-seeking behavior over time to further understand the relationship between intention and future behavior. Additional co-variates of interest may also include variables such as mental health stigma, emotional, financial, and physical barriers to help-seeking, and mental health literacy as components that could impact help-seeking intentions.

### 4.4 Conclusion

Overall findings, similar to previous literature, demonstrate a nuanced story behind the help-seeking intentions of university students and if, in any meaningful way, these intentions are impacted by their current well-being. Findings highlighted, though significant, require the context and co-interpretation from the students themselves to understand how differences in reporting “unlikely” “neither likely nor unlikely” or “likely” to seek help could later impact behaviors of help-seeking. The key takeaway from these findings, for researchers, educators, and administrators who work with university students, is the difference between providers in which students are likely to seek help from. The highest scores across all well-being groups can be found for Intimate Partner, and Personal Relations (factor 1) compared to all other factors. Goodwin’s study (2016) also found that students were mostly likely to seek help from a friend, followed by parent, intimate partner, and then other relative. A preference for those whom students already have a relationship with is clearly demonstrated in different populations across different points in time. This preference for personal relations over professionals is not necessarily a concerning outcome. The recommendation to talk to a friend first could become the “gateway” help-seeking behavior that could then build the confidence and awareness of other professional help-seeking options should there become a need. However, alongside a promotion of help-seeking from personal relations is the need for universal mental health and psychological education. Such education would inform and empower whole communities to support each other and understand signs indicating the potential or necessary escalation of care.

Where the current findings highlight a promising interest in seeking help from personal relations, it also highlights the reluctance to seek help from professional services provided through universities, externally, and through the emerging online and telehealth options. Although initially discouraging, these findings serve as motivation for service providers and those who work with students to improve the bias that may be the cause of such low rates of help-seeking intention. Lacking or inaccurate knowledge about services, lacking awareness about the meaning of mental health and when it is appropriate to seek professional help, and uncertainty around potential consequences of seeking help (including privacy and cost concerns) have all been highlighted in previous literature and are all domains that are changeable and can be improved through tailored and targeted interventions. All recommendations highlighted by these findings focus on a need for mental health and psychological education that is accessible, scalable, and meets the needs of the target community.

## Data availability statement

The datasets presented in this article are not readily available due to privacy. The raw data analyzed in this study and used to support the conclusion of this article will be made available by the authors upon reasonable request. Requests to access the datasets should be directed to Melinda McCabe, melinda.mccabe@monash.edu.

## Ethics statement

The studies involving humans were approved by Monash University Research Ethics Committee (MUHREC). The studies were conducted in accordance with the local legislation and institutional requirements. The participants provided their written informed consent to participate in this study.

## Author contributions

MM: Conceptualization, Data curation, Formal analysis, Investigation, Methodology, Project administration, Resources, Validation, Visualization, Writing – original draft, Writing – review & editing. MB: Formal analysis, Supervision, Writing – review & editing. JG: Supervision, Writing – review & editing. KC: Conceptualization, Methodology, Project administration, Supervision, Writing – review & editing.
