# Supplementary material for: The relationship between university student help-seeking intentions and well-being outcomes
Source: Front Psychiatry. 2024 Jul 5;15:1407689. doi: 10.3389/fpsyt.2024.1407689 (PMC11258002; doi:10.3389/fpsyt.2024.1407689)
Supplement: Supplementary file 1 [file DataSheet_1.docx]

***Supplementary Material***

The relationship between University student help-seeking intentions and wellbeing outcomes.

# APPENDIX A

1. **Statistical output for EFA analyses.**

| Analysis | EFA 1 | EFA 2 | EFA 3 |
| --- | --- | --- | --- |
| Model Parameters | 68 | 62 | 54 |
| Rotation Method | OBLIMIN OBLIQUE | OBLIMIN OBLIQUE | OBLIMIN OBLIQUE |
| Observations (missing) | 178 (1) | 178 (1) | 178 (1) |
| Iterations | 124 | 136 | 84 |
| $X^{2}$ | 26.22 | 20.08 | 53.45 |
| *p-value* | .291 | .217 | .001 |
| *df* | 23 | 16 | 24 |
| *ECVI* | 0.911 | 0.809 | 0.907 |
| *SRMR* | 0.024 | 0.021 | 0.031 |
| *MFI* | 0.991 | 0.989 | 0.921 |
| *CFI* | 0.996 | 0.995 | 0.966 |
| *RMSEA* | 0.028 | 0.038 | 0.083 |

**3 Factor Loadings EFA 1**

| Loadings *(standardized)* | Factor 1 | Factor 2 | Factor 3 | Factor 4 | Factor 5 |
| --- | --- | --- | --- | --- | --- |
| Intimate partner | 0.302 | -0.031 | -0.053 | 0.075 | -0.194 |
| Friend | 0.399* | 0.106 | -0.072 | 0.232 | 0.025 |
| Parent | 0.876* | -0.011 | -0.003 | -0.044 | -0.009 |
| Other Relative | 0.655* | 0.023 | 0.041 | 0.041 | 0.019 |
| University Mental Health Professional | 0.010 | 1.053* | -0.008 | -0.020 | -0.004 |
| External Mental Health Professional | -0.044 | 0.243 | 0.105 | 0.505* | 0.037 |
| Phone or Online Emergency Service | 0.003 | 0.004 | 1.188* | -0.007 | -0.005 |
| University General Practitioner | -0.009 | 0.525* | 0.181 | 0.210 | 0.139 |
| External General Practitioner | 0.004 | -0.016 | -0.022 | 0.944* | -0.014 |
| Minister or Religious Leader | 0.182 | 0.069 | 0.092 | -0.004 | 0.348 |
| Medical Professional through Telehealth* | 0.114 | -0.071 | 0.203 | 0.350* | 0.326 ✝ |
| Mental Health Smartphone Apps | 0.011 | 0.050 | -0.050 | 0.002 | 0.929 |
| Websites or online forums | -0.039 | -0.068 | 0.070 | -0.029 | 0.758 |

* met minimum threshold of .320
✝ loaded onto two factors, highest loading retained

**4 Factor loading EFA 2**

| Loadings | Factor 1 | Factor 2 | Factor 3 | Factor 4 | Factor 5 |
| --- | --- | --- | --- | --- | --- |
| Friend | 0.388* | 0.093 | -0.053 | 0.235 | 0.013 |
| Parent | 0.877* | -0.013 | -0.006 | -0.043 | -0.016 |
| Other Relative | 0.662* | 0.024 | 0.029 | 0.045 | 0.014 |
| University Mental Health Professional | 0.008 | 1.066* | -0.005 | -0.021 | -0.009 |
| External Mental Health Professional | -0.046 | 0.239 | 0.074 | 0.534* | 0.041 |
| Phone or Online Emergency Service | 0.000 | -0.000 | 1.459 | -0.004 | -0.004 |
| University General Practitioner | -0.000 | 0.526* | 0.126 | 0.228 | 0.170 |
| External General Practitioner | 0.006 | -0.021 | -0.017 | 0.927* | -0.021 |
| Medical Professional through Telehealth* | 0.116 | -0.059 | 0.134 | 0.368* | 0.360 ✝ |
| Mental Health Smartphone Apps | 0.012 | 0.057 | -0.036 | 0.004 | 0.887* |
| Websites or online forums | -0.044 | -0.060 | 0.035 | -0.039 | 0.803* |
| Minister or Religious Leader | 0.190 | 0.073 | 0.051 | -0.001 | 0.374* |

* met minimum threshold of .320
✝ loaded onto two factors, highest loading retained

**5 Factor loadings EFA 3**

| Loadings | Factor 1 | Factor 2 | Factor 3 | Factor 4 |
| --- | --- | --- | --- | --- |
| Friend | .409* | 0.225 | 0.036 | -0.014 |
| Parent | .820* | -0.033 | -0.037 | -0.030 |
| Other Relative | .724* | -0.017 | 0.025 | 0.009 |
| University Mental Health Professional | 0.061 | 0.233 | 0.573* | 0.024 |
| External Mental Health Professional | -0.034 | 0.891* | 0.008 | 0.006 |
| Phone or Online Emergency Service | 0.082 | 0.171 | 0.312 | 0.368* |
| University General Practitioner | -0.014 | -0.020 | 1.081* | 0.001 |
| External General Practitioner | 0.076 | 0.563* | 0.158 | 0.012 |
| Medical Professional through Telehealth* | 0.148 | 0.337 | -0.048 | 0.440* |
| Mental Health Smartphone Apps | 0.013 | 0.060 | 0.019 | 0.816* |
| Websites or online forums | -0.066 | -0.070 | -0.009 | 0.843* |
| Minister or Religious Leader | 0.227 | -0.129 | 0.168 | 0.394* |

* met minimum threshold of .320
